# Sex specific retinoic acid signaling is required for the initiation of urogenital sinus bud development

**DOI:** 10.1016/j.ydbio.2014.09.016

**Published:** 2014-11-15

**Authors:** Sarah L. Bryant, Jeffrey C. Francis, Isabel B. Lokody, Hong Wang, Gail P. Risbridger, Kate L. Loveland, Amanda Swain

**Affiliations:** a Division of Cancer Biology, Institute of Cancer Research, 237 Fulham Road, London SW3 6JB, United Kingdom; b Department of Anatomy and Developmental Biology, Clayton, VIC, Australia; c Department of Biochemistry and Molecular Biology, Monash University, Clayton, VIC, Australia

**Keywords:** Sexual differentiation, Prostate initiation, Organogenesis

## Abstract

The mammalian urogenital sinus (UGS) develops in a sex specific manner, giving rise to the prostate in the male and the sinus vagina in the embryonic female. Androgens, produced by the embryonic testis, have been shown to be crucial to this process. In this study we show that retinoic acid signaling is required for the initial stages of bud development from the male UGS. Enzymes involved in retinoic acid synthesis are expressed in the UGS mesenchyme in a sex specific manner and addition of ligand to female tissue is able to induce prostate-like bud formation in the absence of androgens, albeit at reduced potency. Functional studies in mouse organ cultures that faithfully reproduce the initiation of prostate development indicate that one of the roles of retinoic acid signaling in the male is to inhibit the expression of *Inhba*, which encodes the βA subunit of Activin, in the UGS mesenchyme. Through in vivo genetic analysis and culture studies we show that inhibition of Activin signaling in the female UGS leads to a similar phenotype to that of retinoic acid treatment, namely bud formation in the absence of androgens. Our data also reveals that both androgens and retinoic acid have extra independent roles to that of repressing Activin signaling in the development of the prostate during fetal stages. This study identifies a novel role for retinoic acid as a mesenchymal factor that acts together with androgens to determine the position and initiation of bud development in the male UGS epithelia.

## Highlights

• We show that sex specific retinoic acid is required for male UGS bud initiation.
• An increase in retinoic acid can lead to prostate-like formation in females.
• We find that activin repression is a downstream target of RA signalling.
• RA is a novel mesenchymal signal regulating bud initiation along the UGS.

## Introduction

The prostate develops from the urogenital sinus (UGS), an endodermal structure derived from the cloaca. The UGS has a bipotential fate depending on the sex of the embryo and will differentiate into the prostate and bulbourethral gland in males or contribute to the vagina during development but not in the adult in the female (Kurita, 2010, Marker et al., 2003). This sexual differentiation is driven by the presence or absence of androgens. In males, the production of hormones by the fetal testis ensures the development of secondary male specific structures, including the prostate. Two types of tissue are present in the UGS; the epithelium (UGE) and the surrounding mesenchyme (UGM), and prostate development depends on their interaction (Cunha, 2008). The UGM has been shown to specify prostate identity on epithelium from different sources, including bladder (Cunha et al., 1983). Androgens signal through the androgen receptor (AR) and analyses of tissue recombinants with tissue from AR deficient mice have shown that mesenchymal AR is required for prostate formation from the UGS (Cunha and Lung, 1978).

Prostate development is initiated in response to androgens with the UGE budding out and growing into the surrounding mesenchyme. In the mouse this occurs in the fetal period at around E16.5–17.5 (embryonic day 16.5–17.5). The buds then elongate, branch, canalize and cytodifferentiate to become secretory at 12–20 days after birth. The initial steps of bud development are faithfully reproduced in ex vivo organ cultures and these have been used to determine the factors involved in prostate differentiation (Doles et al., 2005, Lopes et al., 1996). However, the molecular pathways involved in prostate development initiation are poorly understood. One factor that has been identified to be important is FGF10, which is produced by the UGM in both sexes (Thomson and Cunha, 1999). Mice that are deficient for FGF10 show rudimentary prostate bud formation that does not progress, even in the presence of androgens (Donjacour et al., 2003).

The vitamin A derivative, retinoic acid (RA), has been implicated in many processes during embryogenesis. In the prostate, RA has been shown to increase prostate budding at embryonic stages but has also shown to inhibit prostate ductal growth and branching at postnatal stages (Aboseif et al., 1997, Seo et al., 1997, Vezina et al., 2008). The levels of the highly biologically active RA are tightly controlled in the embryo in space and time. The dietary form of vitamin A, retinol, is converted to RA in two steps catalyzed by two types of enzymes, the alcohol dehydrogenases, ADH, which are widely expressed, and the aldehyde dehydrogenases, ALDH, with restricted expression patterns. The presence of RA is further restricted by inactivation through metabolizing enzymes such as those belonging to the CYP26 family. RA acts as a ligand for the retinoic acid receptors (RARs) that bind DNA together with the retinoic X receptors (RXRs) at specific retinoic acid response elements (RARE) and contribute to transcriptional control.

In this study we show that RA, synthesized in the male UGM in a sex specific manner, is required for the initial step of prostate formation from the UGS. Addition of RA is able to induce prostate bud formation in the absence of androgens, albeit at reduced potency. Expression and functional analysis revealed that one of the roles of RA is to repress the production of Activin by the UGM that inhibits prostate bud formation. Our study suggests that the combined action of androgen and RA determines early prostate development in the UGE.

## Methods and materials

### Mouse strains

All mouse work was carried out in accordance with the UK Animals (Scientific Procedures) Act 1986. Female MF1 outbred mice were purchased from Harlan UK Ltd. at 6–8 weeks old and mated with male MF1 outbred mice to obtain wild type embryos. For embryo staging, observation of a vaginal plug was designated as embryonic day 0.5 (E0.5). RARE-LacZ mice were generated by Janet Rossant and were kindly provided by Peter McCaffery, University of Aberdeen (Rossant et al., 1991). *Inhba*^−/−^ (*Inhba* mutant) animals lacking the *Inhba* coding sequence were maintained through heterozygote breeding because *Inhba* mutants are neonatal lethal, as previously described (Matzuk et al., 1995).

### UGS organ culture

Fresh UGS tissue was dissected from embryos in PBS by removing the bladder, urethra and ductal tissue using a 5 mm dissection knife, as previously described (Staack et al., 2003). Female UGS were used for all experiments, as they have not been exposed to fetal androgens. Similar organ culture results were also observed when using male UGS, although the degree of prostatic inhibition was variable. This variability was probably due to the presence of older embryos where prostate budding had already initiated at the time of dissection. UGS samples from E15.5 female embryos were chosen because of consistency of bud growth in culture. Similar results were obtained when E14.5 embryos were analysed. To grow tissue caudal to the prostate, the bladder and UGS were identified and the surrounding tissue carefully dissected with forceps until the tissue that will form the bulbourethral gland was located. A dissection knife was then used to remove the prostate and the bulbourethral gland and the intermediate tissue was used for culture. Dissected tissue was grown on 0.4 μm Biopore filters (Millipore, UK) in 2.5 ml of serum-free culture medium (DMEM/Hams F12 1:1) containing 1 x ITS (insulin, transferrin and sodium selenite) (Sigma, UK), 0.025 mg/ml gentamicin (Sigma, UK), 0.06 mg/ml benzylpenicillin sodium, 0.1 mg/ml streptomycin sulphate and 0.05 mg/ml ampicillin. Dihydrotestosterone (DHT) (Sigma, UK) was solubilised in 100% ethanol and added to the media at a concentration of 10^‐8^ M. *All trans* retinoic acid (RA) (10^‐6^ M), the ALDH inhibitor 4-diethylamino-benzaldehyde (DEAB) (50 μM), the pan RAR inverse agonist BMS493 (20 μM) and the activin inhibitor SB431542 (50 μM) were prepared in DMSO and added to the media (Sigma, UK). Control UGS were treated with the equivalent volume of vehicle. The dishes were placed in a humidified incubator at 37 °C in 5% CO_2_ and media was changed at least every 48 h.

### Bud number quantification

Bud number counting was performed on whole mount in situ stained UGS samples or from sections of *Inhba* mutants and controls. Positive buds were defined as those that stained for *Nkx*3.1 (Figs. 2A–C, 4C–E, and 5), were clear bud-like structures on a light microscope (Fig. 2D) or by histological features with haematoxylin and eosin staining (Fig. 4A). Statistical significance when two treatments were compared was calculated using the Student’s two-tailed *T*-test.

### Real-time PCR (RT-PCR)

RNA was extracted from tissue using an RNeasy Micro kit (Qiagen). cDNA was synthesized using OligodT primer and SuperscriptII Reverse Transcriptase (Invitrogen). mRNA accumulation of *Aldh*1*a*1, *Aldh*1*a*2, *Aldh*1*a*3, *Fgf*10, *Inhba* and *Hprt*1 mRNA was determined using the TaqMan system (Applied Biosystems, UK). Triplicates of each sample were analysed. Relative gene expression was calculated using the ΔΔCt method and the housekeeping gene *Hprt*1 as the normaliser. Statistical significance was calculated using the Student’s two-tailed *T*-test.

### Whole mount in situ hybridization analysis

In situ hybridization was carried out on an in situ processor (Intavis In Situ Pro) using a standard protocol as described previously (Val et al., 2006). Digoxigenin-labelled antisense RNA probes for *Aldh*1*a*1, *Aldh*1*a*2, *Aldh*1*a*3 and *Inhβa* were generated from PCR fragments containing T7 RNA polymerase recognition sites using the following primers.

*Aldh*1*a*1*:*5′AGCTCAAGACAGTCGCAATG3′ and
5′GTAATACGACTCACTATAGGGAGTCCTCCTCACCAAATGAG3′
*Aldh*1*a*2*:*5′TGCCAAGACTGCCACGTTTC3′ and 5′GTAATACGACTCACTATAGGGAAGGACTCAAAGCCACTGTC3′
*Aldh*1*a*3*:*5′GGCTAACAAGTAACACCTGG3′ and
5′GTAATACGACTCACTATAGGGCCTCCGTGTACTTACAGCTA3′
*Inhβa:* 5′ATT TGCTGAAGAGGAGAAGG3′ and 5′GTAATACGACTCACTATA GGGCGGCAAAGGTGATGATCTCC3′

Probes for *Nkx*3.1 and *Sox*9 have been described previously (Bhatia-Gaur et al., 1999, Thomsen et al., 2008). Stained whole-mount in situ hybridization samples were fixed in 4% paraformaldehyde, taken through a sucrose gradient before being frozen in OCT (R.A. Lamb) at −80 °C and then sectioned.

### β-Galactosidase stain

Transgenic embryos and organ cultures expressing RARE-LacZ were fixed in 4% paraformaldehyde for 1 h at 4 °C, and then washed in PBS. LacZ stain solution (1 mg/ml X-gal, 5 mM K_3_Fe(CN)_6_, 5 mM K_4_Fe(CN)_6_, 2 mM MgCl_2_ and 0.02% NP40) was applied to the tissue and incubated in the dark at 37 °C until stain was visible. Embryos from the same litter were used for each experiment to control for transgene heterogeneity.

### Immunohistochemistry

Antibody stains were performed on paraffin sections. Tissues were fixed overnight in 4% paraformaldehyde (PFA), dehydrated by washing through an ethanol gradient series, washed in histoclear and embedded in wax. 4 µm sections were cut, treated with histoclear and rehydrated through an ethanol gradient series. Antigen retrieval was obtained by boiling the sections in citrate buffer (0.1 M sodium citrate pH6 and 0.05% Tween). Sections were incubated in PBS with 10% sheep serum and then incubated with primary and secondary antibodies in PBS with 1% sheep serum. Sections were counterstained with DAPI nuclear stain. The following antibodies were used for immunohistochemistry; SOX9 was a kind gift from Francis Poulat (CNRS, Montpellier, France, 1:2000), Ki67 (clone TEC-3, Dako, 1:20), E-Cadherin (BD Transduction Labs, 1:50). Secondary fluorescent antibodies were obtained from Molecular Probes and were used at a 1:500 dilution. Fluorescent images were visualized and collected on a Leica TCS-SP2 confocal microscope.

## Results

### RA is required for the initiation of prostate development from the UGS

**Fig. 1:**
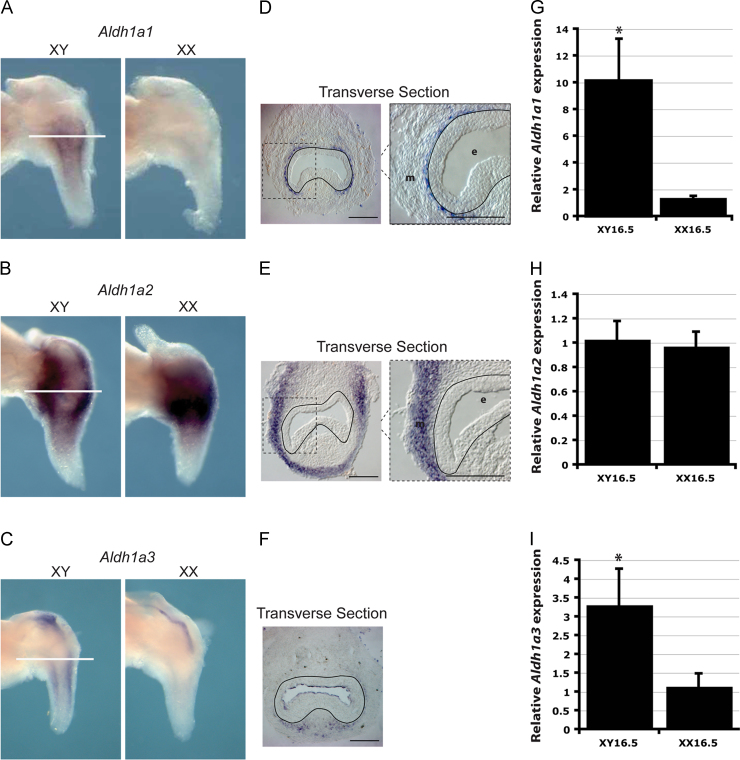
*ALDH* expression in the developing mouse prostate. (A)–(C) Whole mount in situ hybridization analysis of *Aldh*1*a*1, *Aldh*1*a*2 and *Aldh*1*a*3 expression in E15.5 male and female UGS. Images are representative from 3 UGS. (D)–(F) transverse sections through E15.5 male UGS show *Aldh*1*a*1 expression in the periurethral mesenchyme, *Aldh*1*a*2 expression in the mesenchyme and *Aldh*1*a*3 expression in the dorsal and lateral periurethral mesenchyme. A black line marks the basement membrane. (G)–(I) RTPCR confirms the sex-specific expression of *Aldh*1*a*1 in E16.5 male (*p*=0.0006), no difference in expression of *Aldh*1*a*2 between E16.5 male and female UGSs (*p*=0.56) and confirms an increase in expression of *Aldh*1*a*3 in the E16.5 male UGS (*p*=0.005) (*n*=4 for each sample). Error bars=SD. ^⁎^=Statistically significant difference in expression levels. *e*=Epithelia, *m*=mesenchyme. White lines denote planes of section. Scale bar=10 μM. See also Fig. S1.

ALDH enzymes catalyze the final step in RA synthesis and have restricted expression patterns reflecting areas of active signaling. The expression of the ALDH enzymes, *Aldh*1*a*1, *Aldh*1*a*2 and *Aldh*1*a*3, in the mouse UGS has been investigated, although not in both sexes (Vezina et al., 2008). Consistent with previous data, whole mount in situ hybridization analysis showed restricted expression of all three enzymes in the UGM of male embryos at the early stages of prostate development. Interestingly, *Aldh*1*a*1 and, at a lower level, *Aldh*1*a*3 expression were sex specific in that they were undetected in the female UGS at E15.5, while *Aldh*1*a*2 was present in both males and females (Fig. 1A–C). This sex specificity was confirmed by RTPCR (Fig. 1G–I). In the case of *Aldh*1*a*1, we observed induction of expression in female UGS after 24 h, but not 6 or 12 h, of growth in culture in the presence of dihydrotestosterone (DHT) (Fig. S1A, see below for organ culture details). This suggests that induction of *Aldh*1*a*1 is not an immediate early response to androgen action on the UGS. Analysis of sections from stained UGS showed that *Aldh*1*a*1 and *Aldh*1*a*3 had a more peri-urethral expression pattern at the epithelial–mesenchymal boundary within the male UGM, while *Aldh*1*a*2 was expressed in the outer mesenchyme in a zone that may contain smooth muscle cells (Fig. 1D–F) (Thomson et al., 2002).

**Fig. 2:**
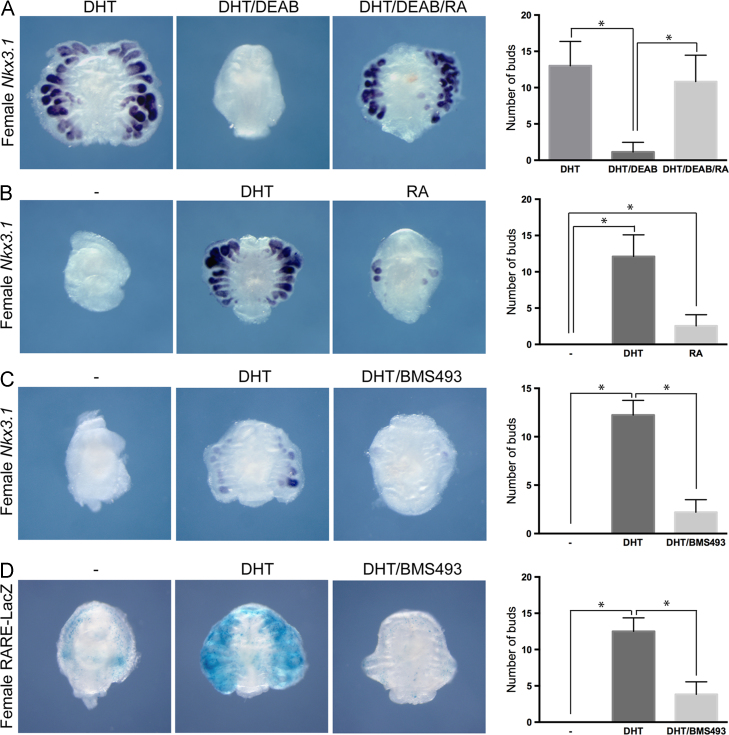
Retinoic acid signaling through RARs is necessary for prostatic bud initiation and can promote budding in the absence of testosterone. (A) Whole mount in situ hybridization analysis of *Nkx*3.1 expression on E15.5 female UGS grown in DHT organ culture for 6 days with and without the ALDH inhibitor DEAB, and with DEAB and RA, as indicated (*n*=13). (B) *Nkx*3.1 expression in E15.5 female UGSs grown in organ culture for 6 days with vehicle (−) (*n*=17), DHT (*n*=15) or retinoic acid (*n*=24), as indicated. (C) Whole mount in situ hybridization analysis of *Nkx*3.1 expression on E15.5 female UGS grown for five days with vehicle (−), DHT in the presence and absence of a RAR inverse agonist, BMS493 (*n*=8). (D) X-gal staining of E15.5 female RARE-LacZ UGS subjected to the same treatment conditions, as indicated (*n*=8). See also Fig. S2. Quantification of bud numbers as described in the Methods and materials section is included for all treatments. ^⁎^=Statistically significant difference in bud numbers.

The expression pattern of ALDH enzymes in the male UGS indicated that RA might have a role in prostate development initiation. This was also suggested by studies performed by Vezina et al. (2008) who showed that addition of RA to UGS cultures grown with DHT increased the number of prostatic buds formed. ALDH enzymes have been known to functionally compensate for each other, therefore an in vivo gene deletion approach was not chosen due to the low feasibility of effectively deleting all three enzymes in the fetal UGM. Instead, we used an *ex vivo* organ culture assay where UGS from E15.5 female mouse embryos were dissected, placed on filters and grown in defined media with and without additional supplements. Addition of DHT induced visible prostate bud formation in 2–3 days of culture and samples were analysed after 5–6 days in culture, at a stage when fully formed buds can be differentiated from transient structures. Whole mount in situ hybridization on cultured female UGS showed expression of *Nkx*3.1 and *Sox*9, early markers of prostate development, in the nascent buds (Bhatia-Gaur et al., 1999, Thomsen et al., 2008). In the absence of DHT, no buds or marker expression were observed. *Nkx*3.1 expression, possibly marking transient prostatic bud structures, has been observed in female UGS grown in culture by others (Allgeier et al., 2010, Keil et al., 2012), however, this was not the case in our conditions. To confirm this result we histologically analysed sections of our samples grown in the absence of drugs and found no evidence of bud-like structures. To determine the role of RA, we cultured female UGS with DHT in the presence of 4-diethylamino-benzaldehyde (DEAB), a well characterised inhibitor of ALDH enzymes, and these showed a severe reduction in *Nkx*3.1 and *Sox*9 expression and bud number (Fig. 2A, see Supporting Fig. S2A for *Sox*9 expression). To establish the specificity of DEAB action, RA was added to the media containing DEAB and DHT and prostate bud development was seen to be activated in these cultured organs (Figs. 2A and S2A). Therefore these data show that RA is required for prostate development from the UGS. To establish if RA is sufficient to initiate prostate development in the absence of DHT, we added RA to the media. Prostate bud development was observed in these tissues after 4 days of culture, however, the number and size of buds was considerably smaller than tissues grown with DHT (Figs. 2B and S2B).

RA has been shown to act through RARs and the expression of RARα, RARβ and RARγ has been observed in the mouse UGS (Vezina et al., 2008). To investigate whether RARs were important in prostate development, we treated female UGS with a pan RAR inverse agonist, BMS493, in the presence of DHT. As in the case of DEAB, BMS493 repressed prostate bud formation, albeit not as effectively, showing that RA was acting through RARs in the UGS (Figs. 2C and SC). To confirm that the compound was acting appropriately, we treated female UGS from RARE-LacZ transgenic embryos expressing LacZ driven by a promoter containing RAREs (Rossant et al., 1991). In untreated and DHT treated female UGS, β-galactosidase staining was observed, with an increase in levels in the DHT treated samples (Fig. 2D). This confirms the presence of active RA signaling within the UGS that is further induced by androgens. Treatment with BMS493 and DHT showed a reduced level of β-galactosidase staining in the UGS, reflecting the inhibiting action of the compound (Fig. 2D). Members of the Cyp26 family of genes have been shown to restrict the pattern of active RA during embryogenesis and expression of *Cyp*26*a*1 and *Cyp*26*b*1 have been reported in the UGS (Vezina et al., 2008). However, we were not able to observe expression above background in the UGS at the early stages for both these genes in either sex. Analysis of β-galactosidase expression in male and female UGS from RARE-LacZ embryos showed staining in both sexes. Although the staining was found to be mosaic and variable in intensity between litters, we did observe an increase in periurethral staining in males compared to females, consistent with the sex specific ALDH gene expression patterns we describe above (Fig. S1B and C).

### RA represses Activin signaling in the UGS

**Fig. 3:**
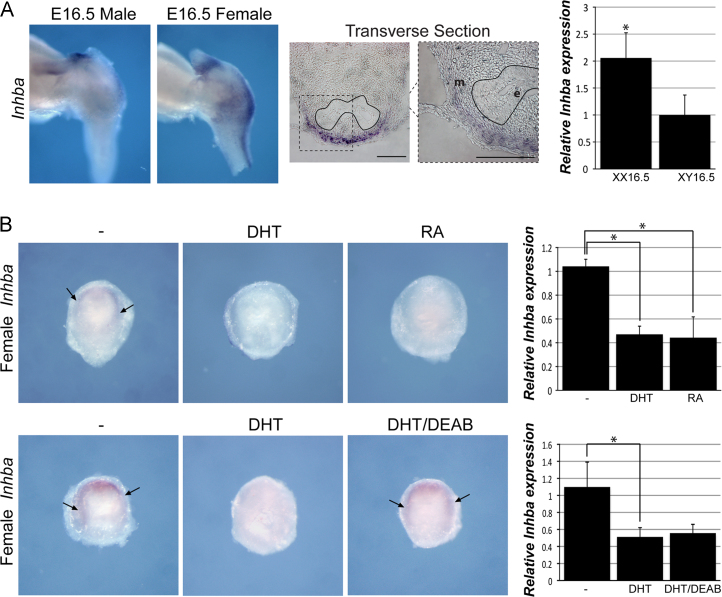
RA represses activin signaling in the UGS. (A) Whole mount in situ hybridization analysis of *Inhba* shows sex-specific expression in E16.5 female UGS compared to male. Adjacent panels, a transverse section showing *Inhba* expression in a female UGS and RTPCR confirms the sex-specific expression of *Inhba* (*p*=0.038). A black line marks the basement membrane on the transverse section. (B) Top panels, whole-mount in situ hybridization analysis for *Inhba* expression on female E15.5 UGS organ cultures grown for 24 h with vehicle (−), DHT or RA, as indicated (*n*=5). Right, RTPCR confirms a decrease in *Inhba* expression in UGS grown 24 h in DHT or RA (no DHT v DHT *p*=0.002, no DHT v RA *p*=0.020) (*n*=3). Bottom panels, *Inhba* expression in E15.5 female UGS organ culture for 24 h grown with vehicle (−), DHT with and without DEAB, as indicated (*n*=5). Right, RTPCR analysis of *Inhba* expression from UGS grown 24 h with vehicle (−), DHT with and without DEAB (*n*=4). Error bars=SD. ^⁎^=Statistically significant difference in expression levels. *e*=epithelia, *m*=mesenchyme.

Our studies show that RA is required for the initiation of prostate development from the UGS. Expression analysis of genes known to be involved in embryogenesis in male and female UGS at E16.5 identified Inhibin βA (*Inhba)* as being higher in female UGS compared to male UGS at this stage (Fig. 3A). This sex difference was confirmed by RTPCR. Interestingly, expression was found to be restricted to the mesenchyme surrounding the UGE with highest levels in the dorsal area (Fig. 3A). *Inhba* encodes the βA subunit of Activin, a member of the Transforming Growth Factor β (TGFβ) family involved in many processes during embryonic development. Activin A, a dimer composed of two βA subunits, has been implicated in prostate morphogenesis and it was shown to inhibit branching when added to organ cultures of rat ventral prostates (Cancilla et al., 2001). To investigate the role of DHT and RA on *Inhba* expression in the UGS, we analysed, by in situ hybridization and RTPCR, female UGS that had been incubated in RA, DHT or DHT and DEAB (Fig. 3B). The in situ hybridization data showed that treatment with DHT and RA led to a striking decrease in *Inhba* levels, while treatment of DHT and DEAB showed the resurgence of *Inhba* in a pattern similar to untreated UGS. The RTPCR studies showed a statistically significant decrease in *Inhba* expression with RA or DHT. However, treatment with DEAB and DHT showed variable levels of *Inhba* expression in the RTPCR analysis that were not statistically significantly different to the DHT treatment when averaged. This difference may be due to variability in amounts of tissue dissected, which has more impact for the RTPCR analysis, or suggests that repression of *Inhba* by DHT can also occur through other non RA dependent mechanisms. Therefore, these data suggest that in the UGS of male embryos RA represses *Inhba* expression and indicates that the inhibitory effect of DHT on this expression is, at least partially, through its action on RA synthesis.

**Fig. 4:**
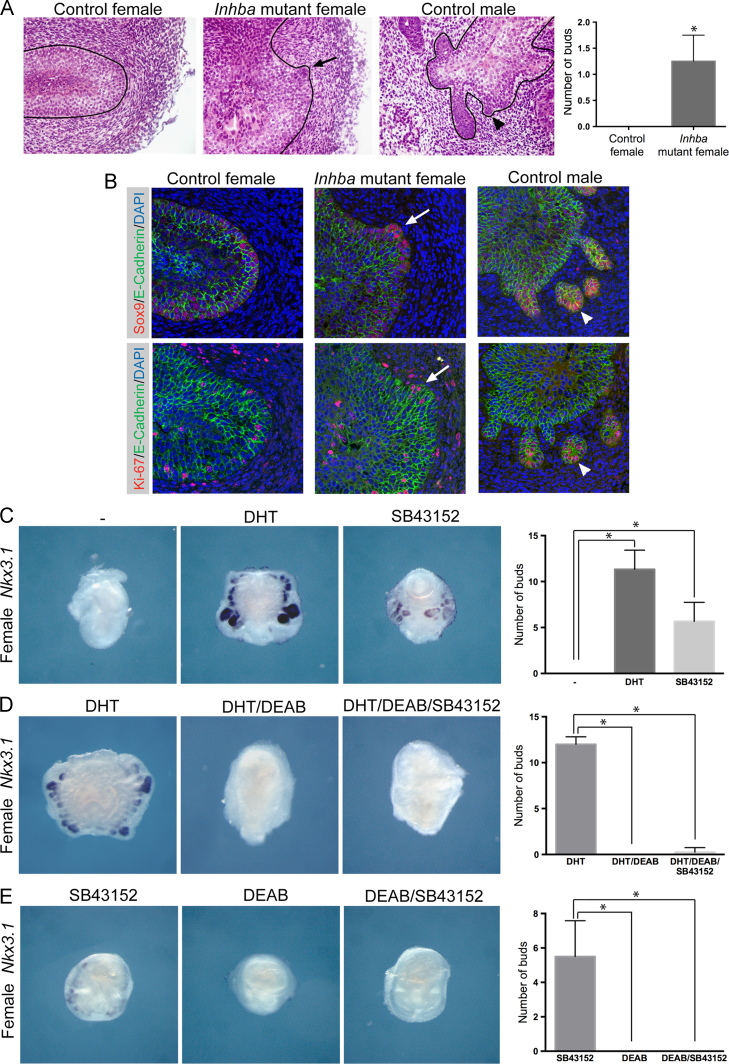
Activin represses prostate formation and RA is required for bud outgrowth. (A) Hematoxylin and eosin stained sections of E18.5 control female UGS, *Inhba* mutant female UGS and control male UGS. An arrow indicates a bud in female *Inhba* mutant and an arrowhead indicates a bud in control male (*n*=4). Black line highlights the epithelial-mesenchymal boundary. Quantification of bud numbers as described in the Methods and materials section is included. (B) Immunohistochemical staining on sections of E18.5 control female UGS, *Inhba* mutant female UGS and control male UGS. Top panels, staining of Sox9 (red) and E-Cadherin (green), show high levels of Sox9 in female *Inhba* mutant buds (white arrow) and control male buds (white arrowhead). Bottom panels, staining of Ki67 (red) and E-Cadherin (green), show Ki67 positive cells in female *Inhba* mutant buds (white arrow) and control male buds (white arrowhead). E-Cadherin staining marks the epithelial cells of the UGS. (C) Whole mount in situ hybridization analysis of *Nkx*3.1 expression in E15.5 female UGS organ cultures grown for 6 days with vehicle (−), with DHT or the inhibitor of TGFb type I receptors, SB43152, as indicated (*n*=5). (D) *Nkx*3.1 expression in E15.5 female UGS organ cultures grown in DHT for 6 days with and without DEAB and SB43152, as indicated (*n*=5). E, *Nkx*3.1 expression in E15.5 female UGS organ cultures grown for 6 days with SB43152, DEAB or both SB43152 and DEAB, as indicated (*n*=5). Quantification of bud numbers as described in the Methods and materials section is included for all treatments. ^⁎^=Statistically significant difference in bud numbers. (For interpretation of the references to color in this figure legend, the reader is referred to the web version of this article.)

To investigate the functional role of *Inhba* we used in vivo genetic analysis in addition to *ex vivo* organ culture assays. As *Inhba* mutant mice that lack functional protein die within 24 h of birth, we studied mutant embryos at E18.5 (Matzuk et al., 1995). Strikingly, female *Inhba* mutant UGS at this stage contained small epithelial buds that protrude in the surrounding mesenchyme similar to immature buds found in male UGS (Figs. 4A and S3A, 4 out of 4 sectioned mutant female UGS showed at least one prostatic bud-like structure but no buds were observed in the 5 female control UGS analysed). No buds were observed in control female UGS. These female *Inhba* mutant buds expressed high levels of Sox9 and Ki67, a marker of proliferating cells, in a similar pattern to control male buds (Fig. 4B). This demonstrates that mutant female UGS initiate the early stages of prostate-like budding in the absence of *Inhba*. However, these structures are smaller and fewer than observed in normal males indicating that additional pathways, some of which probably involving androgens, are required for bud elongation and growth. In complimentary experiments, we treated female UGS with SB43152, an inhibitor of TGFβ type I receptors, including the Activin type I receptor, ALK4. In agreement with our genetic studies, after 3–4 days of culture early prostatic-like buds were observed in female UGS treated with the inhibitor in the absence of DHT (Figs. 4C and S3B). This phenotype was more pronounced than the effect of RA treatment, however, the number and size of buds were generally smaller than those derived from DHT treated female UGS (Fig. 4C). These data are consistent with the model that Activin represses prostate formation from the UGS and RA acts to relieve this inhibition and allow early bud development. To determine if the only role of RA in the UGS is to inhibit Activin signaling, we investigated if SB43152 treatment was able to induce bud formation in female UGS treated with DHT and DEAB. Interestingly, the Activin inhibitor did not phenocopy RA in that it was not able to induce prostate-like tissue in the presence of DHT and DEAB (Figs. 4D and S3C). Consistent with this result, female UGS that had been treated with both the Activin and ALDH inhibitors in the absence of DHT did not show prostatic-like buds (Figs. 4E and S4D). These data therefore show that RA has additional roles to the inhibition of Activin signaling in the induction of prostate development from the UGS.

To investigate whether RA has a role in prostate development after the initial steps of bud induction, we treated male UGS from later stage (E17.5–E18.5) embryos with DHT or DHT and DEAB. Tissues treated with DEAB showed a reduced number and smaller sized buds when compared to samples treated with DHT, while samples without added compounds showed the presence of very small buds confirming that bud induction had taken place before treatment (Fig. S4). This effect is overcome when RA is added to media with DHT and DEAB (Fig. S4).

FGF10, which is expressed in the outer region of the UGM, is required for the initial stages of prostate development (Donjacour et al., 2003) and RA and FGF signaling have been shown to interact in the morphogenesis of other organs such as the lung (Desai et al., 2004). This suggests that FGF10 may be a target of RA signaling in the UGS. Therefore we investigated whether RA signaling can regulate FGF10 expression in the UGS prior to prostate budding. However, RTPCR analysis on female UGS samples treated with RA, DHT or DHT and DEAB showed no consistent changes in FGF10 levels (Fig. S5).

### RA and DHT induce bud development in non-prostatic UGE

**Fig. 5:**
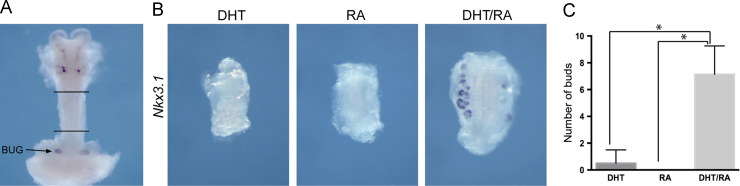
RA and DHT induce bud development in non-prostatic UGE. (A) The region of the male urethra that was dissected and grown in culture. Whole mount in situ hybridization analysis of *Sox*9 expression carried out on a E18.5 prostate, urethra and bulbourethral gland (BUG). Black lines indicate the urethra. (B) Whole mount in situ hybridization analysis of *Nkx*3.1 expression in male tissue dissected caudally, between the prostate and the bulbourethral gland, grown in organ culture for 6 days with DHT, RA or both DHT and RA (*n*=9). Quantification of bud numbers as described in the Methods and materials section is included for all treatments. *=Statistically significant difference in bud numbers. See also Fig. S6.

Prostate development occurs at the cranial end of the UGS, and the pathways that determine this positional information have not been determined. We noticed that the *Aldh*1*a*1 and *Aldh*1*a*2 expression patterns tended to be concentrated at the cranial portion of the UGS (see Fig. 1A and B). Therefore we investigated the potential of RA and/or DHT to induce prostate bud formation in more caudal regions of the UGS. For this, we dissected and grew the area of the male embryonic UGS found between the prostate and the bulbourethral gland, where the epithelium is a continuous tube and no buds are normally found (Fig. 5). Treatment of this tissue with either DHT or RA had no effect. However, treatment with both DHT and RA showed the formation of *Sox*9 and *Nkx*3.1 positive early bud-like structures after 4 days in culture (Figs. 5 and S6A).

## Discussion

**Fig. 6:**
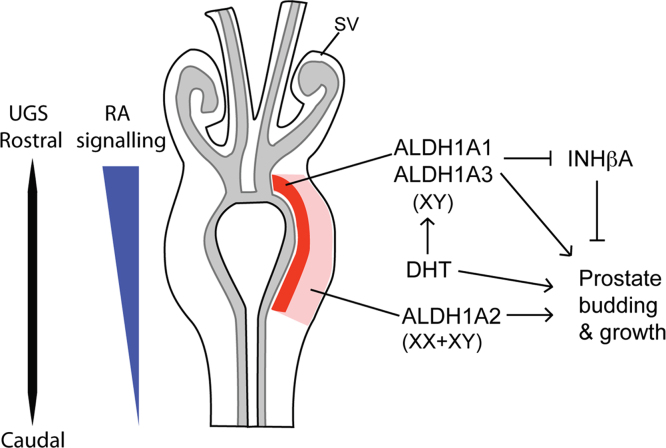
Model depicting RA action in the UGS. Representation of an embryonic UGS with epithelial (grey) and mesenchymal (red) regions. *Aldh*1*a*2 expression is shown in the outer mesenchyme (light red) in both males and females and *Aldh*1*a*1 and *Aldh*1*a*3 are male specific and in more peri-urethral regions (darker red). We speculate that in the male UGS, DHT action leads to high RA levels that inhibit *Inhba* expression and promote prostate budding and growth. SV=seminal vesicle (blue). (For interpretation of the references to color in this figure legend, the reader is referred to the web version of this article.)

Very little is known on how the prostate arises from the UGS in male, but not female embryos. The central role of androgens in this process has been known for some time, however, the mechanism of action has not been elucidated. In this study we show that RA signaling is required for prostate formation in both an androgen dependent and independent manner. The expression pattern of ALDH enzymes in the UGS and our functional studies suggest a model where androgen action in the male leads to an increase in RA levels in the peri-urethral region of the UGM that brings about the decrease in levels of *Inhba* and therefore the relief of prostate formation inhibition by Activin (Fig. 6). Our results are also consistent with both RA and androgens having extra independent roles, in addition to the regulation of Activin, in the early stages of bud growth. These data identify RA as a major player in the initiation of prostate development, together with androgens.

In vivo studies have shown that all ALDH enzymes are able to induce RA synthesis (Fan et al., 2003). In addition, we have observed the presence of *Cyp*1*b*1, which has been shown to generate RA independently of ALDH (Chambers et al., 2007), within the periurethral UGS mesenchyme of male but not female embryos (Fig. S6B). Therefore a genetic approach to manipulate this pathway was not feasible. Our data indicate that the ALDH1A2 present in the female UGS is not able to inhibit *Inhba* expression. In contrast to previous studies (Vezina et al., 2008), we were not able to observe significant expression in the mouse UGS of members of the Cyp26 family, *Cyp*26*a*1 and *Cyp*26*b*1, suggesting that active RA is present in the female. This is in agreement with the β-galactosidase positive staining the female UGS of the RARE-LacZ mice (Fig. S1). Therefore our results are consistent with amount of active RA within the UGM being important in determining the initiation of prostate development in a sex specific manner.

Prostatic-like buds have been observed in some female UGS of the rat (Thomson et al., 2002, Timms et al., 1999), although we never observed this in the mice we analysed. In humans, the female paraurethral or Skene׳s glands have been proposed to be the histological homolog of the male prostate (Huffman, 1948). Our studies suggest that investigation into the levels of RA and/or vitamin A in the human female UGS during embryonic development may provide information on the presence of these structures and help explain the species difference. Our studies show that prostate-like development occurs in UGS from *Inhba* mutant female mice, and we did not observe the presence of *Inhbb*, which can compensate for some aspects of *Inhba* function (Brown et al., 2000). This suggests that the levels of Activin in the UGS during embryogenesis could also affect this outcome.

The UGM has been proposed to specify prostate identity on epithelium derived from various tissues and on embryonic stem cells when recombined and grown as kidney grafts (Cunha et al., 1983, Taylor et al., 2006). Androgens and AR are required for this process, however, our studies show that DHT alone is not able to induce bud formation at ectopic sites in the UGS but that a combination of DHT and RA is required. ALDH expression in the mouse UGS is concentrated in the cranial region of the UGS, where prostate development occurs, and is highest during fetal stages and decreases significantly around birth. This suggests that RA contributes to the competence of the UGM to specify prostate formation by providing a regional and stage context.

RA signaling has been identified to be important in many inductive processes during embryonic development. In lung morphogenesis, it has been shown to inhibit TGFβ signaling in the endodermal derived foregut to allow FGF10 expression and primary lung bud formation (Chen et al., 2007). However, Activin expression was not investigated in this pathway and we found no evidence of a relationship between RA and FGF10 in the UGS. Therefore, it is likely that the downstream pathways regulated by RA in the embryo are dependent on the tissue.

Our study identifies a role for RA during the initiation of prostate formation from the UGS. Although we observe that RA is required for prostate growth at later stages, ALDH expression in the UGM decreases with age. ALDH1a1 has been shown to be expressed in adult human prostate epithelia and ALDH1a2 has been detected in adult prostate epithelia of mouse and man (Kim et al., 2005, Li et al., 2010, Touma et al., 2009). This suggests a complex role for this signaling pathway as the prostate differentiates and matures. Consistent with this, RA treatment of neonatal prostates can inhibit morphogenesis and growth (Aboseif et al., 1997). Retinoids, including RA, have been proposed as antitumour agents in prostate cancer and epidemiological studies have suggested that consumption of vegetable derived vitamin A could protect against this disease (Pienta and Esper, 1993). However, treatment of human prostate cancer patients with retinoids have demonstrated limited success (Hammond et al., 2002). The data presented could inform the understanding of the effect of retinoids in prostate cancer and suggest that the contribution of stromal RA signaling should also be considered to determine the full role of this important pathway in normal and neoplastic tissue.

## Footnotes

Appendix A Supplementary data associated with this article can be found in the online version at doi:10.1016/j.ydbio.2014.09.016.
