# Supplementary material for: Sex specific retinoic acid signaling is required for the initiation of urogenital sinus bud development
Source: Dev Biol. 2014 Nov 15;395(2):209–17. doi: 10.1016/j.ydbio.2014.09.016 (PMC4211671; doi:10.1016/j.ydbio.2014.09.016)
Supplement: Supplementary file 1 — Supplementary data. [file mmc1.pdf]

Supporting figure legends

Figure S1. A, RTPCR analysis of *Aldh1a1* mRNA levels in E15.5 female UGS grown with (+) or without (-) DHT for the time periods indicated (n=4). Error bars=SD. *= significant difference in expression levels (p=0.00003). B, X-gal staining for β-galactosidase activity in the UGS of male and female E15.5 mice carrying the RARE-LacZ transgene. C, X-gal staining for β-galactosidase activity in transverse sections of the UGS of male and female E17.5 mice carrying the RARE-LacZ transgene. White arrows indicate high X-gal staining at the periurethral region. Black line marks the epithelial-mesenchymal boundary.

Figure S2. A, whole mount in situ hybridization analysis of *Sox9* expression on female E15.5 UGSs grown in prostate organ culture for 6 days with DHT, with and without the ALDH inhibitor DEAB, and with DEAB and RA, as indicated (n=12). B, *Sox9* expression in female E15.5 UGSs grown in prostate organ culture for 6 days with DHT (n=15), vehicle (-) (n=17) or retinoic acid, as indicated (n=24). C, whole mount in situ hybridization analysis of *Sox9* expression in E15.5 female UGS grown for 5 days with vehicle (-), with DHT in the presence and absence of a RAR inverse agonist, BMS493 (n=4).

Figure S3. A, Low magnification image of hematoxylin and eosin stained sections of E18.5 control female UGS, *Inhba* mutant female UGS and control male UGS. A black arrow indicates a bud in a female *Inhba* mutant and a white arrow indicates a bud in a control male (n=4). Black line highlights the epithelial-mesenchymal boundary. Magnification is 20x. B, whole mount in situ hybridization analysis of *Sox9* expression on E15.5 female UGS prostate organ cultures grown for 6 days with vehicle (-), with

DHT or the inhibitor of TGFb type I receptors, SB43152, as indicated (n=5). C, *Sox9* expression in E15.5 female UGS prostate organ cultures grown in DHT for 6 days with and without DEAB and SB43152, as indicated (n=5). D, *Sox9* expression on E15.5 female UGS prostate organ cultures grown for 6 days with SB43152, DEAB or both SB43152 and DEAB, as indicated (n=5).

Figure S4. A, *Nkx3.1* expression in E17.5 male UGS grown in prostate organ culture for 5 days with vehicle (-), DHT, and DHT and DEAB, with or without RA as indicated (n=8). B, *Sox9* expression in E17.5 male UGS grown in prostate organ culture for 5 days with DHT, and with and without DEAB and RA, as indicated (n=6).

Figure S5. RTPCR analysis for *Fgf10* expression of female E15.5 UGS prostate organ cultures grown for 24 hours with vehicle (-), DHT, RA, or DHT and DEAB, as indicated. No significant difference in *Fgf10* expression was observed (top chart; no DHT v DHT p=0.124, no DHT v RA p=0.525, bottom chart; no DHT v DHT p=0.101, no DHT v DHT/DEAB p=0.242) (n=3). Error bars = SD.

Figure S6. A, whole-mount in situ hybridization analysis of *Sox9* expression in tissue dissected caudally, between the prostate and the bulbourethral gland, grown in organ culture for 6 days with DHT, RA or both DHT and RA (n=5). B, whole-mount in situ hybridization analysis of *Cyp1b1* expression in E18.5 female and male UGS. Right panel, a transverse section through E18.5 male UGS shows expression in the periurethral mesenchyme.

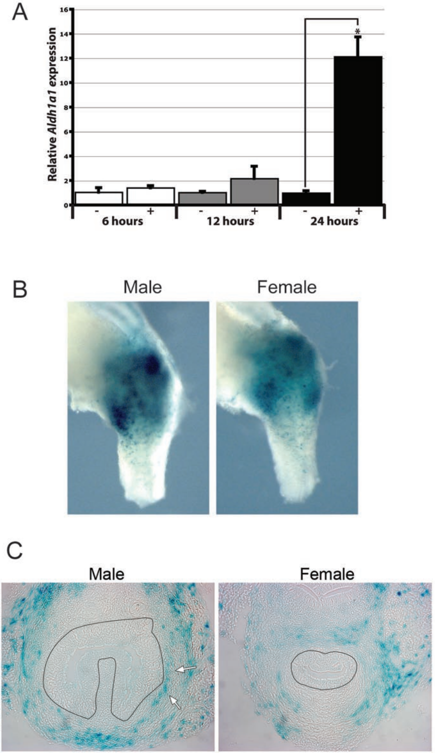

A
Relative Aldh1a1 expression
0
2
4
6
8
10
12
14
16
*
-
+
6 hours
12 hours
24 hours
B
Male
Female
C
Male
Female
E17.5 RARE-LacZ

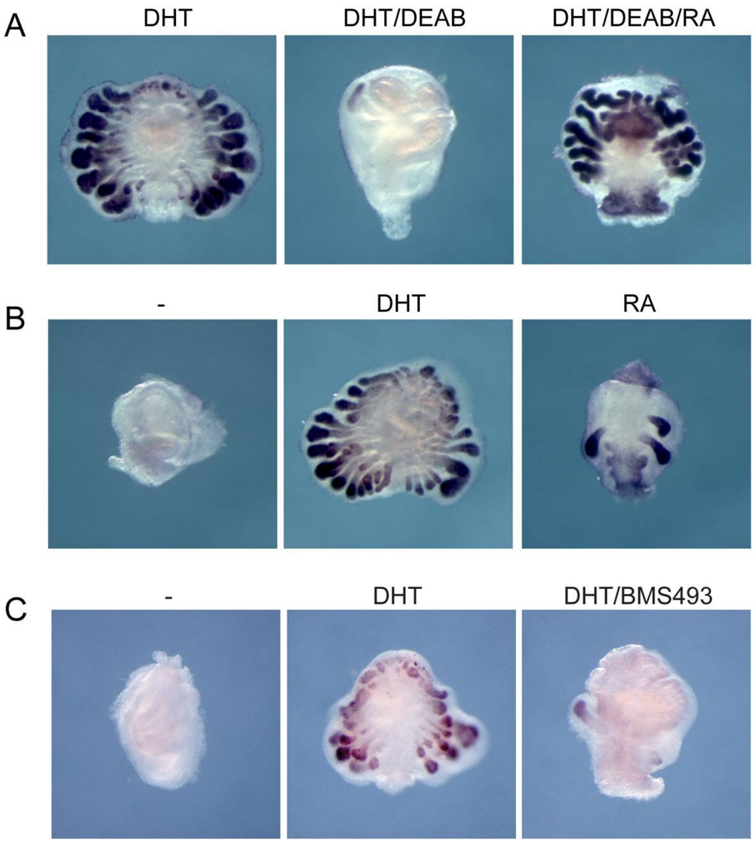
A
DHT
DHT/DEAB
DHT/DEAB/RA
B
-
DHT
RA
C
-
DHT
DHT/BMS493

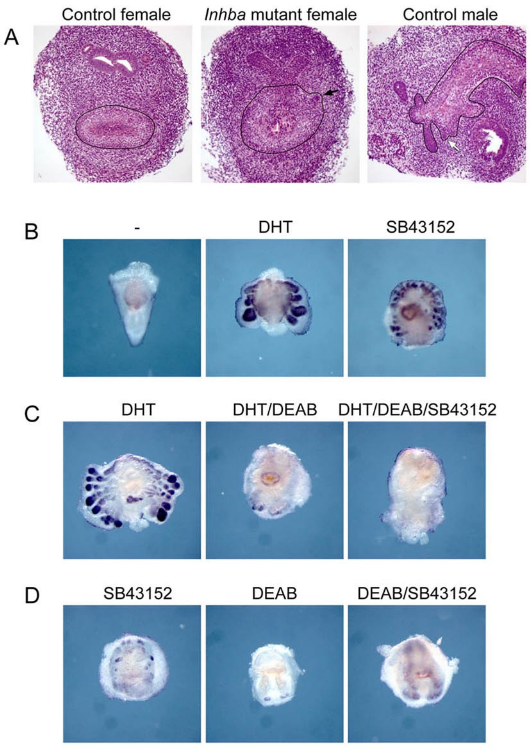
A
Control female
Inhba mutant female
Control male
B
-
DHT
SB43152
C
DHT
DHT/DEAB
DHT/DEAB/SB43152
D
SB43152
DEAB
DEAB/SB43152

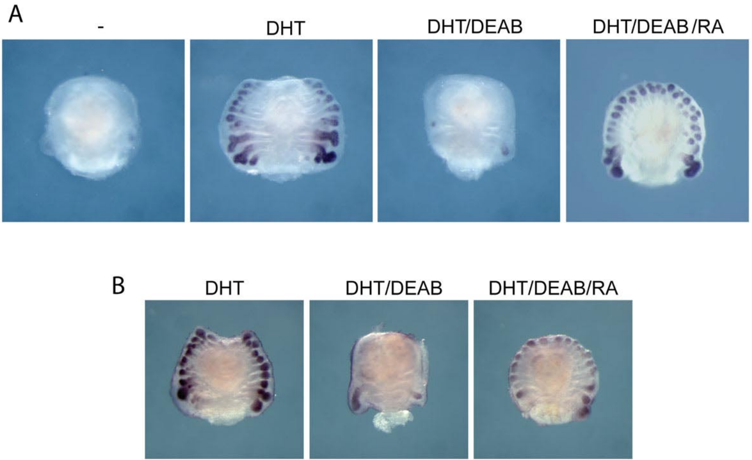

A
-
DHT
DHT/DEAB
DHT/DEAB/RA
B
DHT
DHT/DEAB
DHT/DEAB/RA

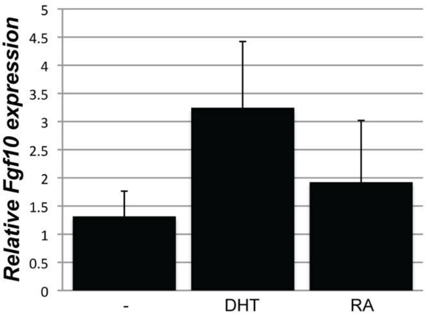

Relative Fgf10 expression
5
4.5
4
3.5
3
2.5
2
1.5
1
0.5
0
-
DHT
RA

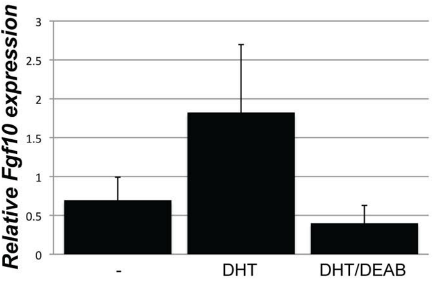

Relative Fgf10 expression
3
2.5
2
1.5
1
0.5
0
-
DHT
DHT/DEAB

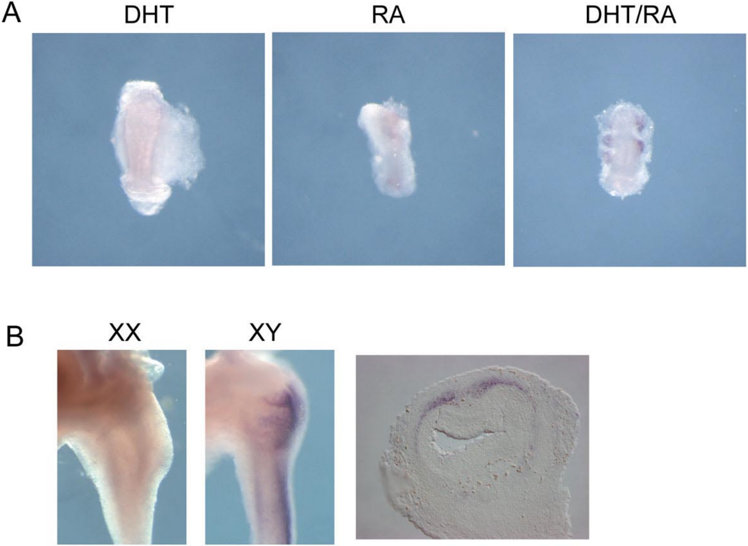
A
DHT
RA
DHT/RA
B
XX
XY
